# Supplementary figures and images for: FUS regulates RAN translation through modulating the G-quadruplex structure of GGGGCC repeat RNA in C9orf72-linked ALS/FTD
Source: eLife. 2023 Jul 18;12:RP84338. doi: 10.7554/eLife.84338 (PMC10393046; doi:10.7554/eLife.84338)

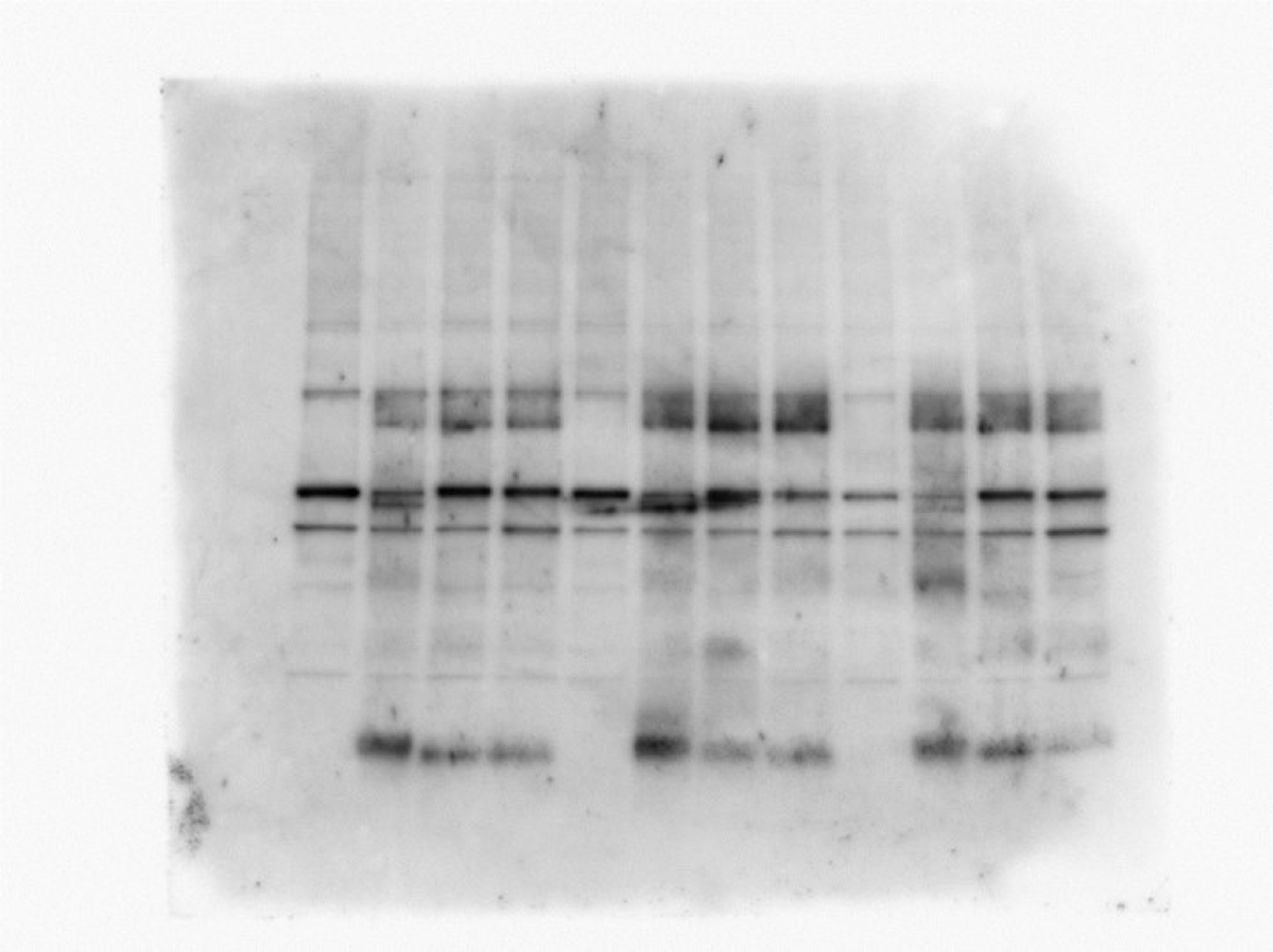

Supplement: Figure 2—figure supplement 1—source data 2. [file elife-84338-fig2-figsupp1-data2.zip › Figure 2 figure supplement 1 source data 2/FUS, actin/FUS and action raw.jpg]

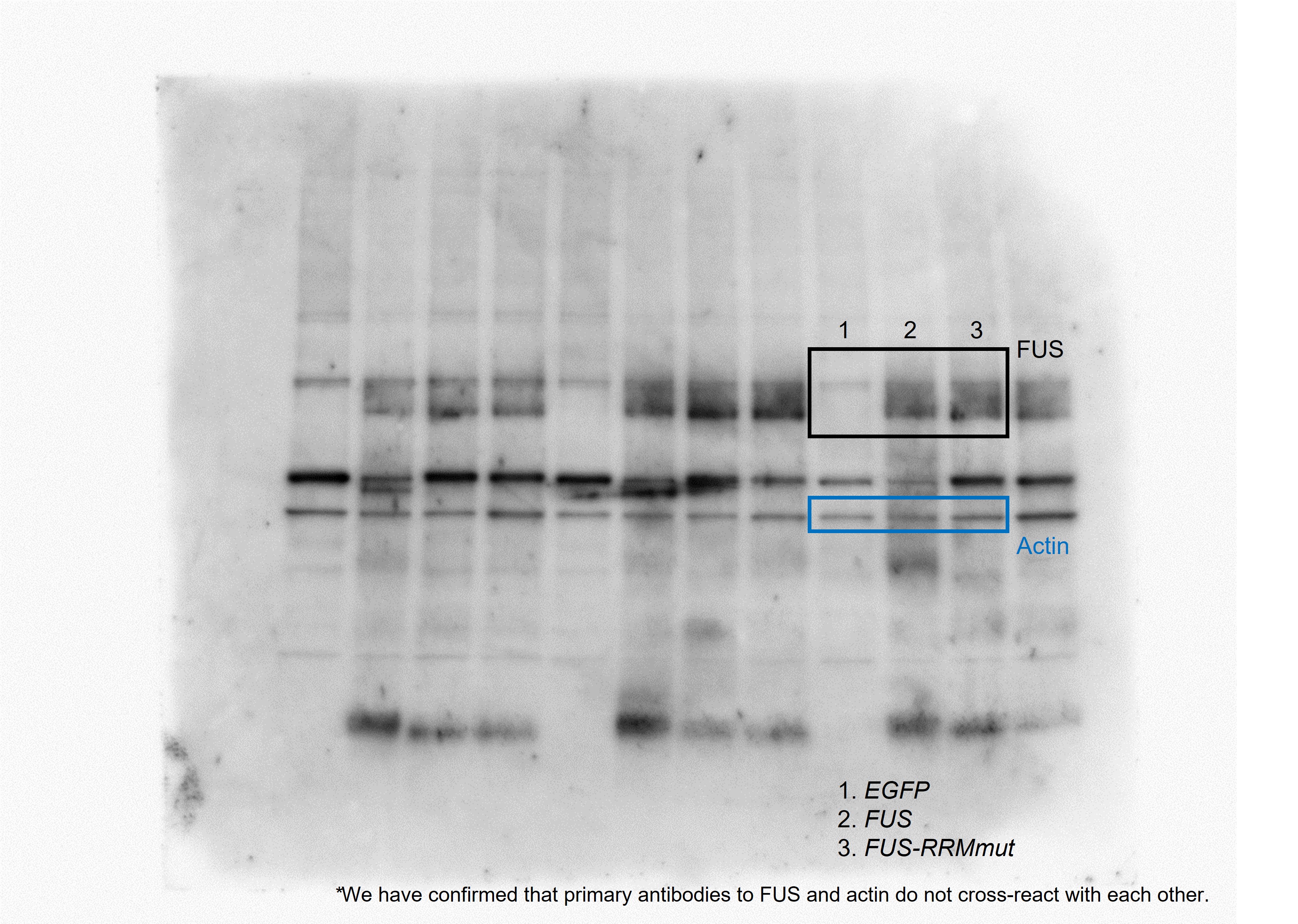

Supplement: Figure 2—figure supplement 1—source data 2. [file elife-84338-fig2-figsupp1-data2.zip › Figure 2 figure supplement 1 source data 2/FUS, actin/FUS and action with labels.jpg]

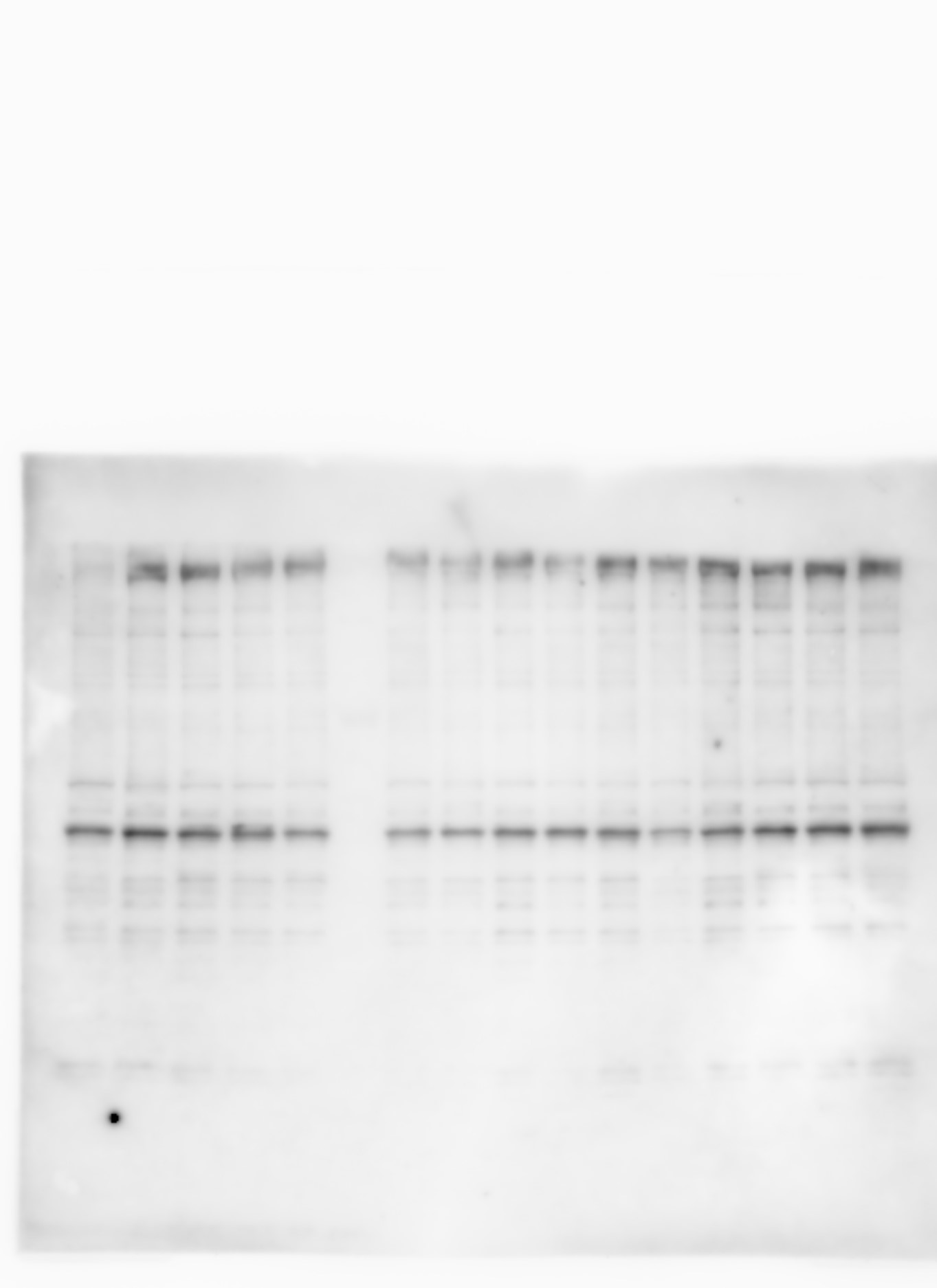

Supplement: Figure 3—source data 2. [file elife-84338-fig3-data2.zip › Figure 3 source data 2/actin/Actin raw.jpg]

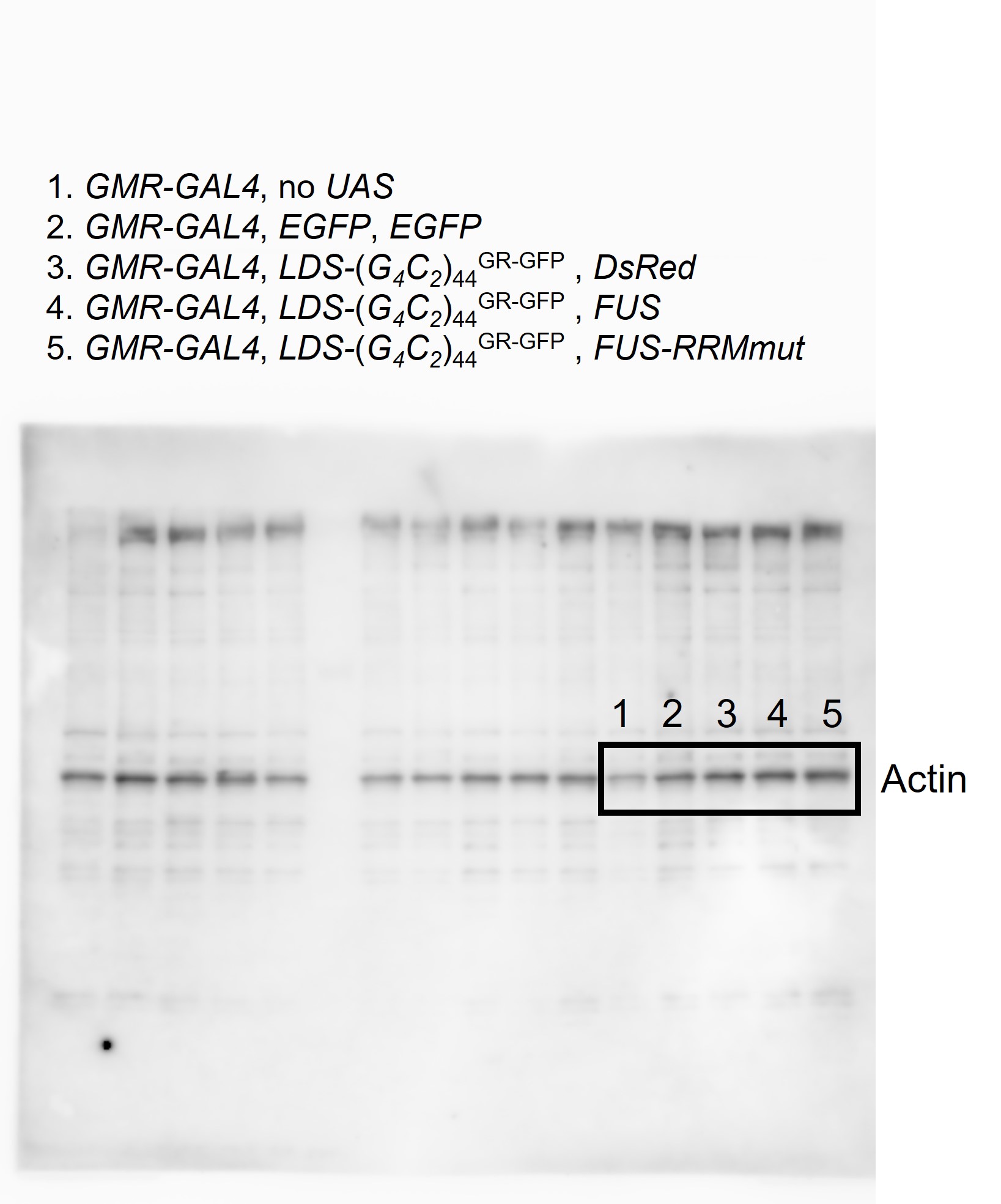

Supplement: Figure 3—source data 2. [file elife-84338-fig3-data2.zip › Figure 3 source data 2/actin/Actin with labels.jpg]

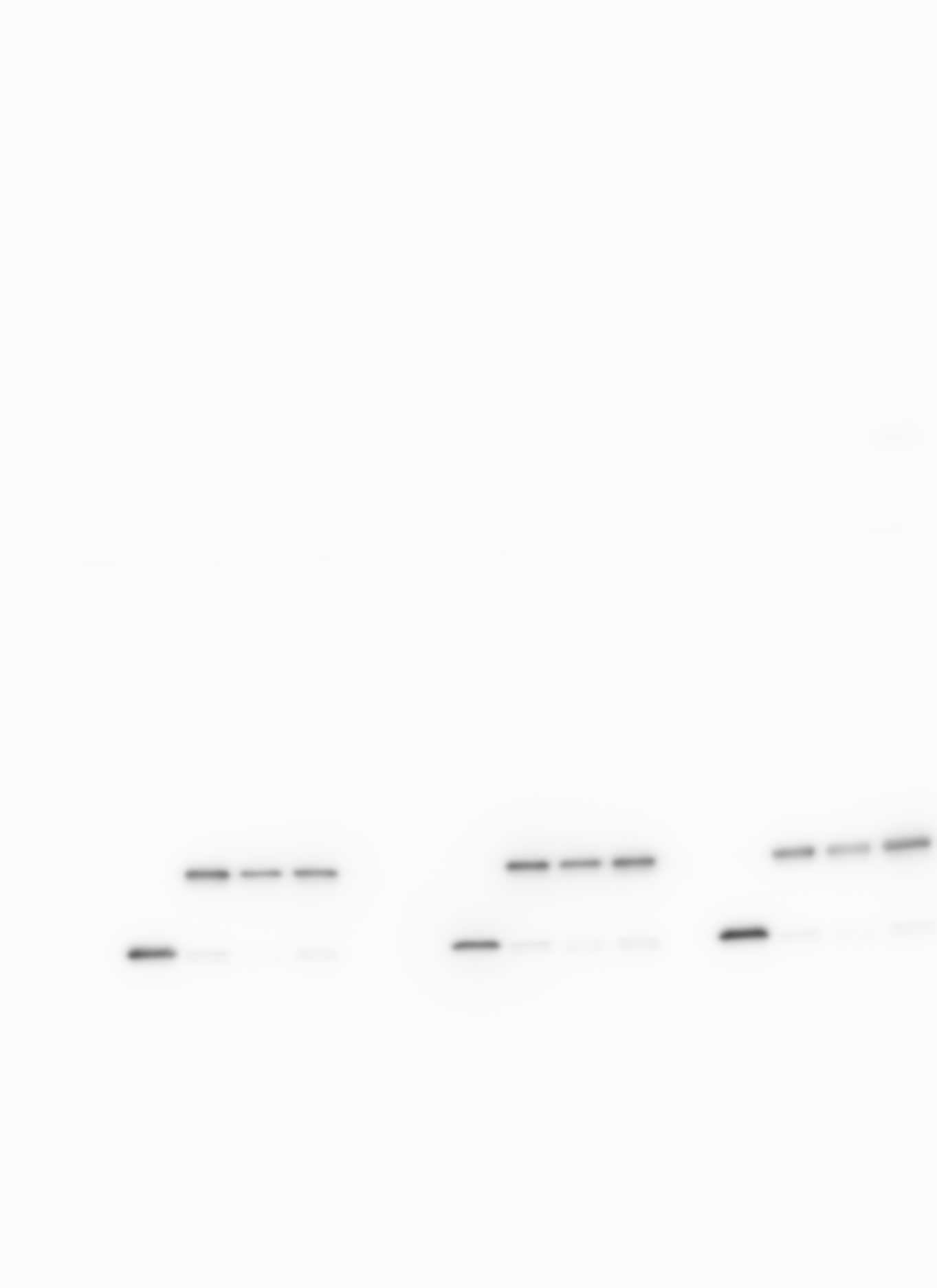

Supplement: Figure 3—source data 2. [file elife-84338-fig3-data2.zip › Figure 3 source data 2/EGFP/EGFP raw.jpg]

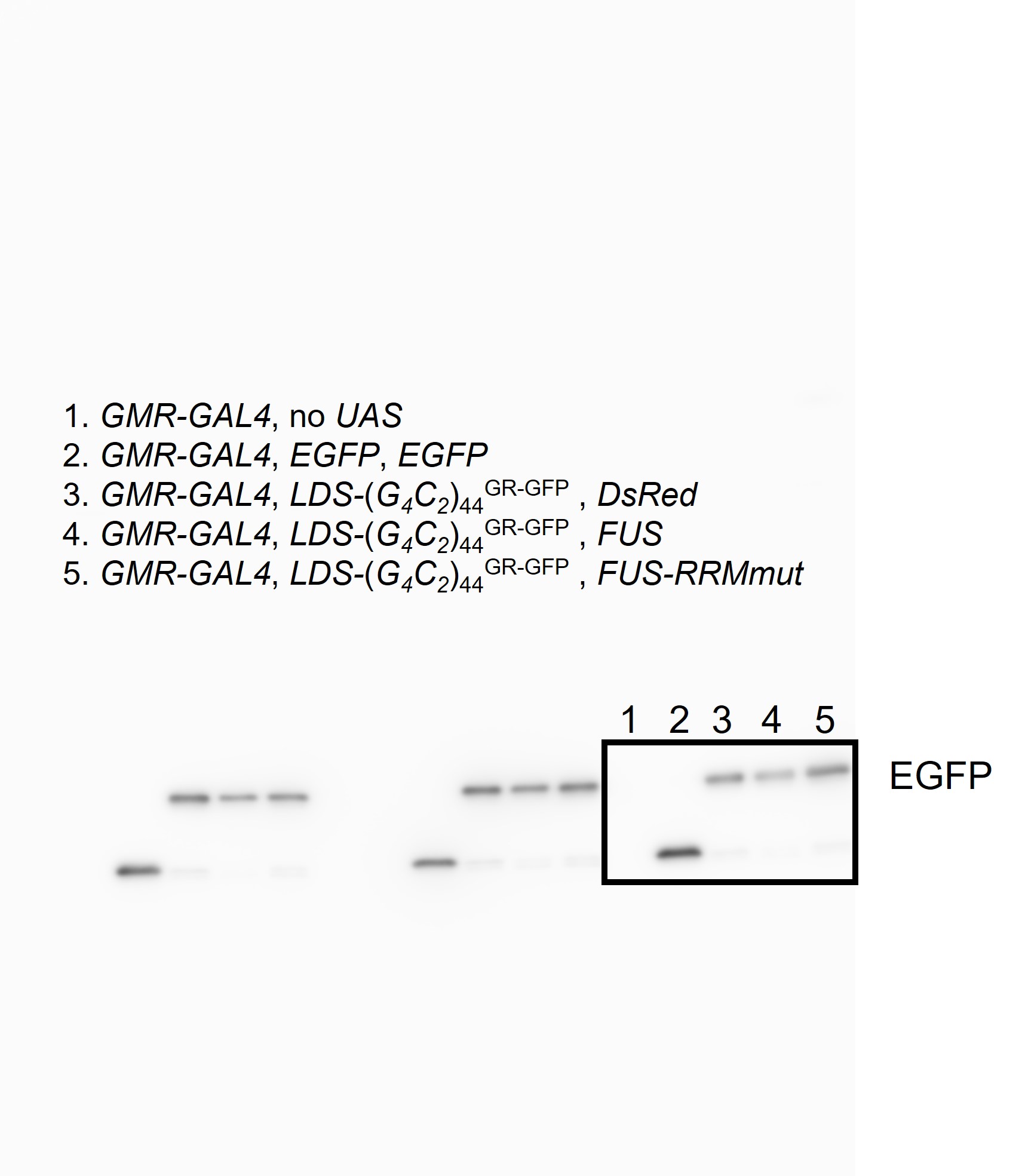

Supplement: Figure 3—source data 2. [file elife-84338-fig3-data2.zip › Figure 3 source data 2/EGFP/EGFP with labels.jpg]

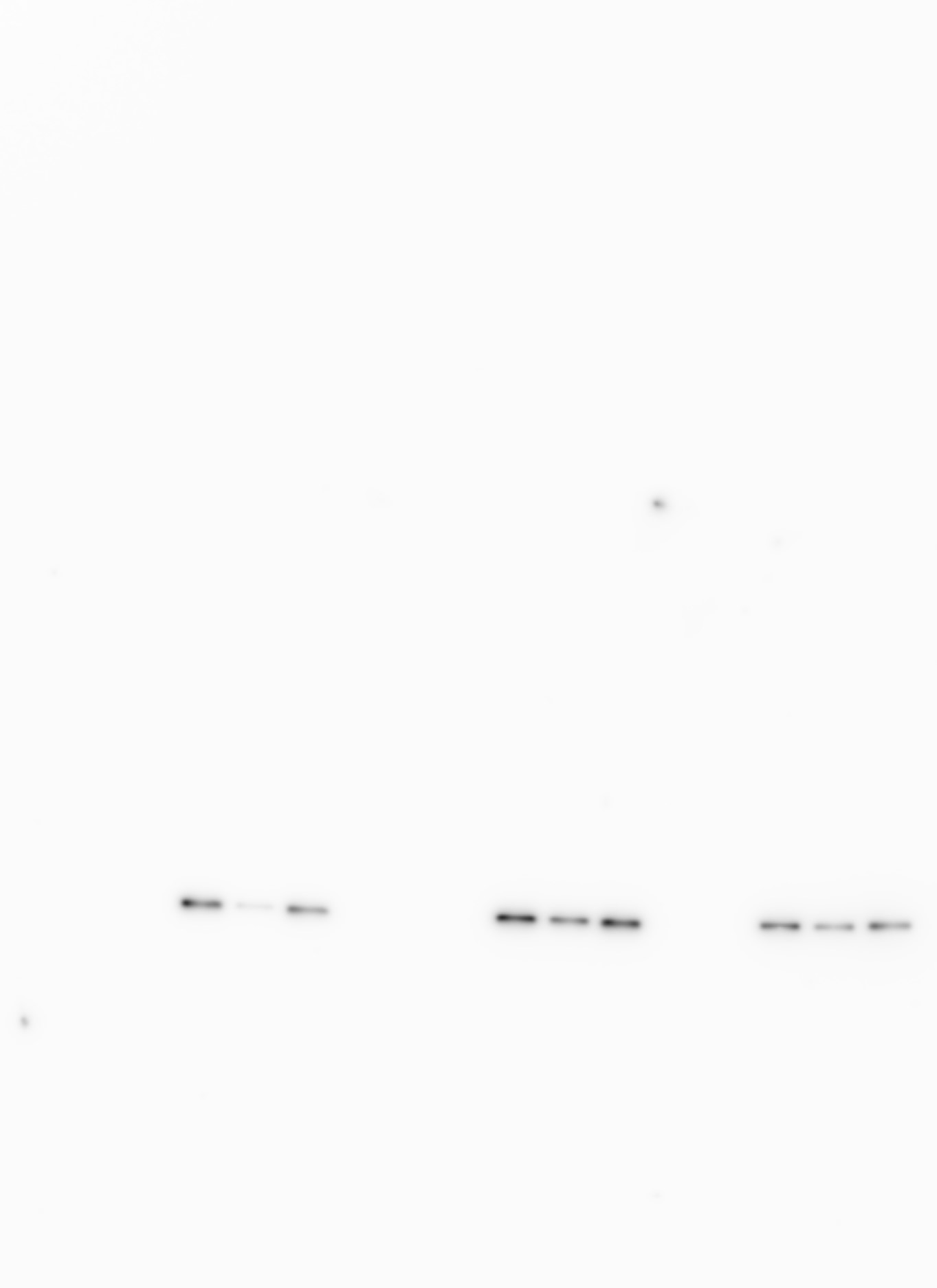

Supplement: Figure 3—source data 2. [file elife-84338-fig3-data2.zip › Figure 3 source data 2/GR/GR raw.jpg]

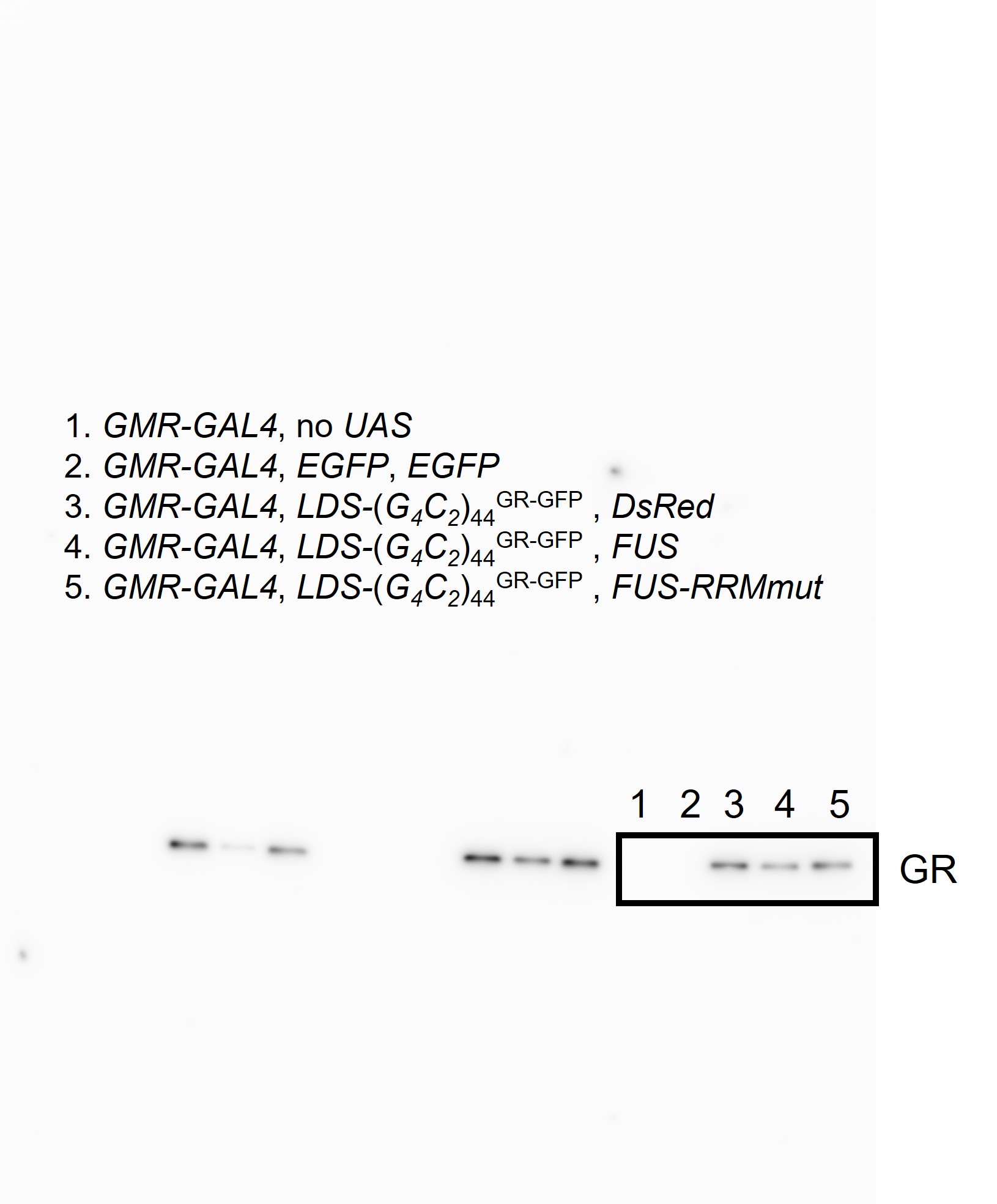

Supplement: Figure 3—source data 2. [file elife-84338-fig3-data2.zip › Figure 3 source data 2/GR/GR with labels.jpg]

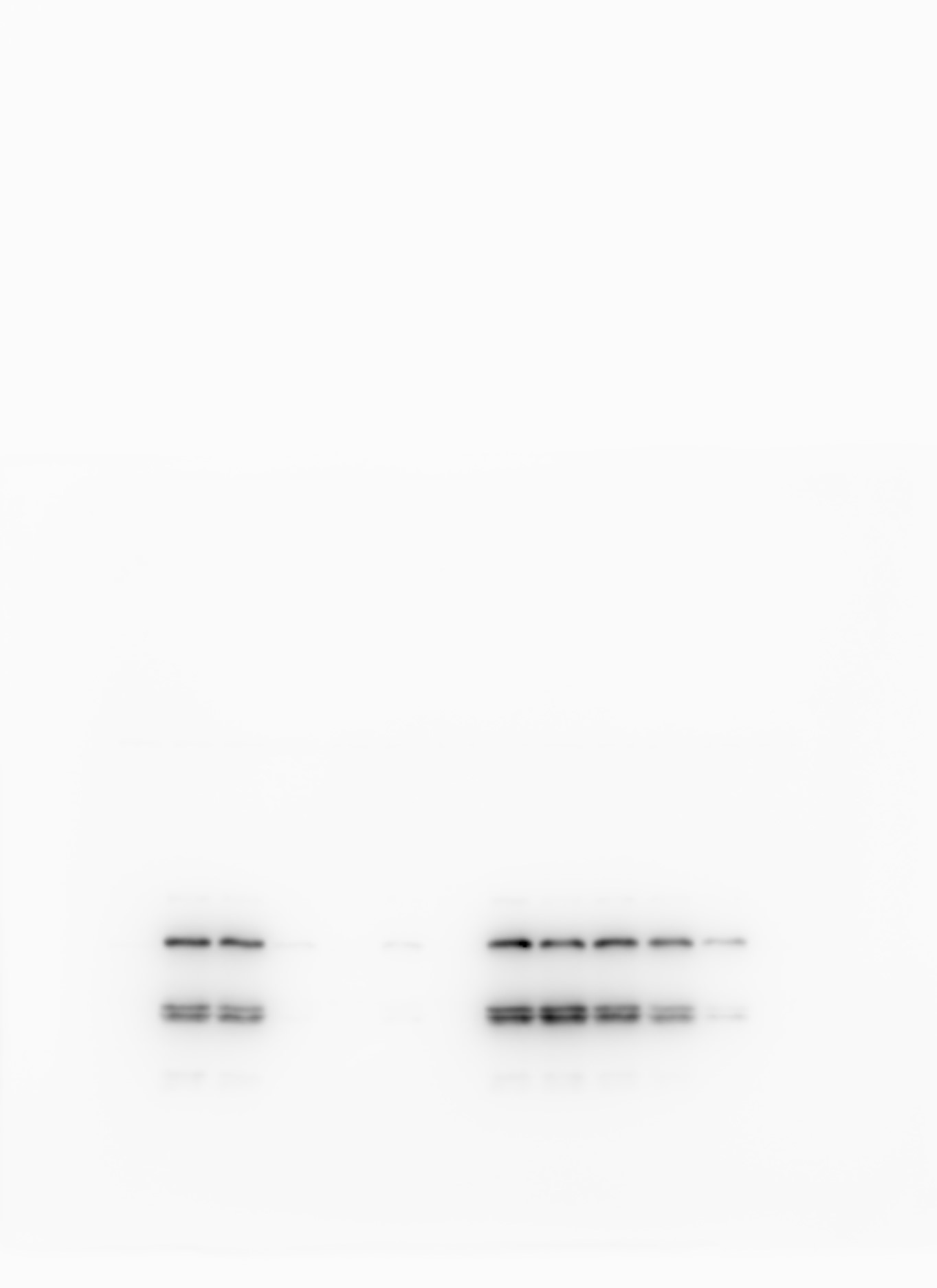

Supplement: Figure 5—source data 2. [file elife-84338-fig5-data2.zip › Figure 5 source data 2/Myc/GA-Myc raw.jpg]

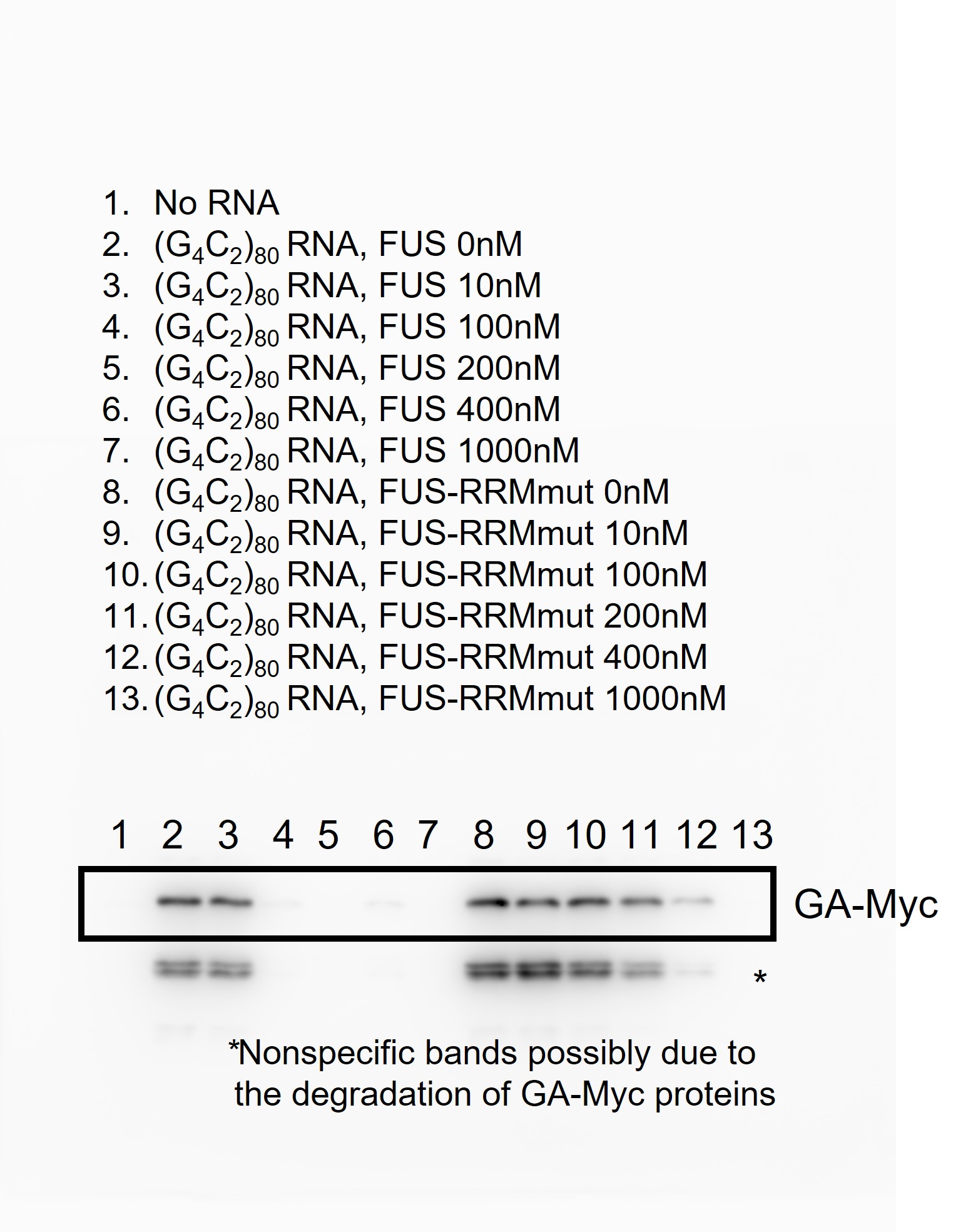

Supplement: Figure 5—source data 2. [file elife-84338-fig5-data2.zip › Figure 5 source data 2/Myc/GA-Myc with labels.jpg]
